# Programmable Reflection–Transmission Shared‐Aperture Metasurface for Real‐Time Control of Electromagnetic Waves in Full Space

**DOI:** 10.1002/advs.202100149

**Published:** 2021-05-26

**Authors:** Lei Bao, Qian Ma, Rui Yuan Wu, Xiaojian Fu, Junwei Wu, Tie Jun Cui

**Affiliations:** ^1^ Institute of Electromagnetic Space Southeast University Nanjing 210096 China; ^2^ State Key Laboratory of Millimeter Waves Southeast University Nanjing 210096 China; ^3^ Institute of Intelligent Metamaterials Pazhou Laboratory Guangzhou 510330 China

**Keywords:** full‐space modulation, independent reflected and transmitted phase modulations, programmable metasurfaces

## Abstract

Recently, programmable metamaterials or metasurfaces have been developed to dynamically edit electromagnetic waves for realizing different functions in the same platform. However, the proposed programmable metasurfaces can only control reflected or transmitted wavefronts in half‐space. Here, a “Janus” digital coding metasurface with the capabilities to program various electromagnetic functions in the reflected (with R‐codes) and transmitted (with T‐codes) waves simultaneously is presented. Three PIN diodes are employed to design the metaparticle, and the state of the PIN diodes can be switched to change the reflected and transmitted phases independently. Three schemes achieved by the proposed programmable metasurface are provided as illustrative examples, including anomalous deflections, beam focusing, and scattering reduction in the full space. As a proof‐of‐concept, a prototype composed of 10 × 20 metaparticles is fabricated and the measured results are in good agreement with the designs and numerical results, validating the full‐space modulations enabled by the programmable metasurface. It is expected that the new programmable metasurface can broaden the applications in stealth technologies, imaging systems, and the next generation of wireless communications.

## Graphical abstract

A “Janus” coding programmable metasurface is proposed to edit the reflected and transmitted waves simultaneously in real‐time. Benefiting from the design of the separated current paths of diodes, the reflected and transmitted phases can be controlled independently in different polarizations. The proposed metasurface can implement various electromagnetic functions in the reflected (with R‐codes) and transmitted (with T‐codes) regions simultaneously.

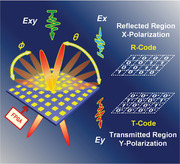

## 1 Introduction

During the past 20 years, metamaterials have experienced an upsurge in a wide variety of scientific and engineering disciplines due to their powerful and tailorable capabilities that are not accessible in nature.^[^ ^1^, ^2^, ^3^, ^4^ ^]^ In general, metamaterials composed of subwavelength artificial structures have the capabilities to manipulate electromagnetic (EM) waves in novel ways effectively. By exploiting these unparalleled EM properties, various physical effects, devices, and applications have been enabled by the metamaterials.^[^ ^5^, ^6^, ^7^ ^]^ Evolving from the metamaterials, metasurfaces are referred to their 2D planar versions and possess many attractive features such as easy fabrication, low profile, and low loss.^[^ ^8^, ^9^, ^10^, ^11^, ^12^, ^13^, ^14^ ^]^ In 2011, the generalized Snell's laws were put forward by Yu et al., revealing the mechanism to manipulate wavefronts by providing abrupt phases on the metasurfaces.^[^ ^8^ ^]^ Since then, the metasurfaces have experienced exponential developments, thereby creating many versatile devices from the microwave to visible light. Although a large number of metasurfaces have been reported so far, most of them are passive, leading to fixed functionalities and characteristics. Aiming to offer the dynamic capabilities for EM manipulations, active and tunable metasurfaces have been proposed and exploited, which could offer a flexible platform for implementing diverse applications and devices.^[^ ^15^, ^16^, ^17^ ^]^

More recently, the concepts of digital coding and programmable metasurfaces have been proposed, providing a digital perspective for controlling the EM wave propagation in real‐time.^[^ ^18^ ^]^ Compared to the metasurfaces with continuous parameters, the digital coding metasurfaces describe the physical parameters (e.g., amplitude, phase, and polarization) in a digital manner with binary codes. Benefiting from the digitalization of physical parameters, the digital coding metasurfaces enable the simple design and binary‐coded manipulations of the EM waves.^[^ ^18^, ^19^ ^]^ For instance, the convolution operation has been applied on metasurface to steer the scattering beams arbitrarily in the upper space with little distortion.^[^ ^19^ ^]^ Moreover, the concept of information entropy was also employed on the digital coding metasurfaces, demonstrating the entropy of the coding patterns and scattering patterns.^[^ ^11^ ^]^ It is worth stressing that the digital coding metasurfaces lay the foundation for the combination of physical and digital sciences. In the microwave region, programmable metasurfaces were initially proposed based on digital coding metasurfaces to enable the realization of dynamic control for the reflected patterns in real‐time, in which PIN diodes are incorporated into the passive meta structures and controlled by field‐programmable gate arrays (FPGAs).^[^ ^18^ ^]^ So far, various active elements, including the PIN diodes, varactors, and photodiodes,^[^ ^20^ ^]^ are used to design the programmable metasurfaces and attain the dynamic modulations of parameters via external stimulus.^[^ ^21^, ^22^ ^]^

Fundamentally, programmable metasurfaces essentially work as wave‐based information systems with different coding sequences and establish a link between the physical and digital worlds. In ref. ^[^ ^23^ ^]^, space‐time‐coding digital metasurfaces were developed to manipulate the spatial beams and frequency spectra simultaneously based on space and time modulations of reflection coefficients. Smart metasurface with self‐adaptively reprogrammable functions in ref. ^[^ ^24^ ^]^ can offer the ability to sense ambient environments by integrating an additional sensor and can adaptively adjust its operational functionality through an unmanned sensing feedback system. Besides, a machine‐learning imager based on the programmable metasurface has been reported in the microwave frequency, which can produce high‐quality imaging and high‐accuracy object recognition directly.^[^ ^25^ ^]^ Many other devices and applications based on the programmable metasurfaces have also been explored by modulating the EM waves arbitrarily and dynamically, such as programmable metasurface holograms,^[^ ^26^ ^]^ single‐sensor imaging,^[^ ^27^ ^]^ and new‐architecture wireless communication systems.^[^ ^28^, ^29^, ^30^ ^]^ Obviously, the recent trends of metasurfaces have advanced toward accomplishing real‐time and programmable functions, but most of the reported works controlled the reflected waves and a few of them controlled the transmitted waves. That is to say, they can only operate in the transmitted or reflected configuration.

On the other hand, with the rapid development of metasurfaces, full‐space control has been one of the steadily increasing interests due to owning great potentials of multiple EM functions for metasurfaces.^[^ ^31^, ^32^, ^33^, ^34^, ^35^, ^36^, ^37^, ^38^ ^]^ In general, the existing metasurfaces have achieved the capability to control both transmitted and reflected wavefronts by changing the polarization, frequency, and direction of the incident wave. Meanwhile, many multiple metadevices and distinct functions have been realized by these metasurfaces to work in both transmitted and reflected modes. For example, in ref. ^[^ ^33^ ^]^, the transmission–reflection integrated bifunctional and trifunctional metasurfaces have been reported with multilayer structures in microwave frequencies. A single‐layer bifacial metasurface has been achieved in the visible region to manipulate the EM waves in full space, which can implement holographic image generation.^[^ ^39^ ^]^ However, the metasurfaces mentioned above with the full‐space controls have fixed functions and cannot reprogrammable, limiting the application potentials.

Here, we present a programmable metasurface operating in the transmitted and reflected modes simultaneously by changing the polarization state of incident waves. For obtaining distinct phase values of the reflected and transmitted waves, we load three PIN diodes on the metaparticle to alter the equivalent circuit by switching the supply voltages of diodes controlled by FPGA. To some extent, the bright prospects of the proposed programmable metasurface establish independent controls in the full space and achieve a flexible platform to sufficiently realize the real‐time modulations of reflected and transmitted EM waves. For digitizing the phase responses, the reflected phases are encoded as R‐codes with digits 0 and 1, while the transmitted phases are encoded as T‐codes with digits 0′ and 1′, respectively. When the reflected and transmitted coding sequences are predesigned, the EM waves on both sides of the metasurface can be excited and modulated separately on demand in orthogonal polarizations. First, we present the fundamental theory and design details of the programmable metaparticle for providing independent controls of the phase responses in the reflection and transmission modes. Then, as a proof of concept, we numerically exhibit that the programmable metasurface can implement various functions, including the beam deflection, diffuse scattering, and beam focusing. Finally, we fabricate a sample composed of 10 × 20 particles and 3 × 10 × 20 PIN diodes for experimental measurements. The measured results are in excellent agreement with the designs and numerical results, validating the performance of the proposed metasurface. We believe that this work can enrich independent modulations and multiple functions of the programmable metasurfaces and promote more potentials in many applications, such as signal processing systems, imaging systems, and smart devices in the next generation wireless communication systems.

## 2 Result

### 2.1 Design of Programmable Metasurface

**Figure 1:**
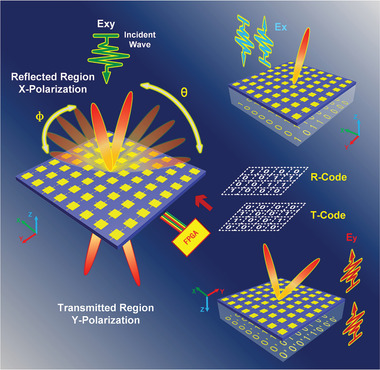
Conceptual illustration of the proposed programmable metasurface with both transmitted and reflected modulations independently. The reflected and transmitted phase responses are determined by the PIN diodes in *x*‐ or *y*‐direction separately. By arranging specific reflected and transmitted coding sequences, the *x*‐polarized reflected beams and *y*‐polarized transmitted beams can be modulated by the programmable metasurface, respectively. When the incidence waves are in oblique polarization, the designed reflected and transmitted beams can be formed simultaneously.

**Figure** **1** shows a schematic diagram of the proposed programmable metasurface to achieve manipulation of EM waves on both sides of the interface. For controlling the transmitted and reflected beams at microwave frequencies, a metaparticle, consisting of rectangle patches and PIN diodes, is designed to modulate the reflected phase responses in the *x*‐polarization and transmitted phase responses in the *y*‐polarization independently with the different bias voltages of diodes controlled by FPGA. When the PIN diodes embedded along the *x*‐axis work on the ON (or OFF) state, the reflected phase response of the metaparticle is encoded as 1 (or 0) under the *x*‐polarized linearly incident waves. Similarly, with or without the PIN diodes embedded along with the *y*‐axis enabling, the transmitted phases response is encoded as 1′ (or 0′) under the *y*‐polarized incidence, respectively. Consequently, when the predesigned transmitted and reflected coding sequences are distributed on the programmable metasurface simultaneously, the metasurface can perform the corresponding functions in the reflection and transmission modes separately. According to the principle to calculate the scattering pattern of the digital coding metasurface consisting of *M* × *N* particles, the scattering pattern in far filed can be written as^[^ ^18^ ^]^

(1) E(θ,ϕ)=∑n=1N∑m=1MA(m,n)exp−iφ(m,n)+kDsinθm−12cosϕ+n−12sinϕ×m−12cosϕ+n−12sinϕ

where *θ* and *ϕ* are the elevation and azimuth angle of the direction, respectively. *A*(*m*,*n*) and *φ*(*m*,*n*) denote the amplitude and phase of the (*M*th, *N*th) metaparticle. *D* is the periodicity of the metaparticle. Based on the polarization synthesis theory,^[^ ^22^ ^]^ the ***E*** (*θ*, *ϕ*) can be decomposed into *x*‐polarized and *y*‐polarized components, *E_x_* (*θ*,*ϕ*) and *E_y_* (*θ*,*ϕ*). Hence, the ***E*** (*θ*, *ϕ*) can be expressed as

(2) E(θ,ϕ)=∑n=1N∑m=1MAx(m,n)exp−iφx(m,n)+kDsinθm−12cosϕ+n−12sinϕ×m−12cosϕ+n−12sinϕex+∑n=1N∑m=1MAy(m,n)exp−iφy(m,n)+kDsinθm−12cosϕ+n−12sinϕ×m−12cosϕ+n−12sinϕey=Ex(θ,ϕ)ex+Ey(θ,ϕ)ey=Er(θ,ϕ)ex+Et(θ,ϕ)ey

The *A_x_* (*m*,*n*), *A_y_* (*m*,*n*), *φ_x_* (*m*,*n*), and *φ_y_* (*m*,*n*) present the amplitude and phase responses of metaparticle in the *x*‐ and *y*‐polarizations, respectively. For the proposed metasurface, the reflected and transmitted phases can be controlled by the *x*‐ and *y*‐polarized illuminations independently. Thus, when we ignore the cross‐polarization components, *E_x_* (*θ*,*ϕ*) and *E_y_* (*θ*,*ϕ*) will be the scattering patterns of the metasurface in reflection and transmission, *E*_r_(*θ*,*ϕ*) and *E*_t_(*θ*,*ϕ*), as shown in Equation (2). We should note that the reflected (*E_x_*) and transmitted (*E_y_*) patterns can be excited and modulated simultaneously under the illumination of the incident waves in oblique polarizations.

**Figure 2:**
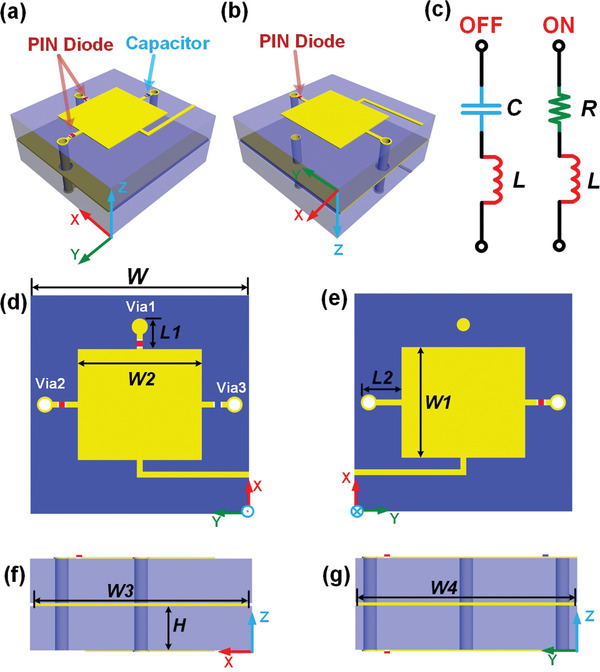
Geometry of the programmable metaparticle. a,b) Perspective views of the proposed programmable metaparticle. c) Equivalent circuit models of the PIN diodes in the OFF and ON states. d–g) Detailed structure of the proposed programmable metaparticle, in which the PIN diodes and capacitor are integrated on the top and bottom surface.

As shown in **Figure** **2**, we design a programmable metaparticle integrated with PIN diodes (M/A‐COM MADP‐000907‐14020x) to realize the aforementioned characteristics. The geometry of the metaparticle is a sandwich‐like structure, consisting of three metal layers and two substrate layers (F4B, with a dielectric constant of 2.65 and loss tangent of 0.001), as illustrated in Figure 2a,b. Two metal patches on the top and bottom layers are printed on the substrate layers, respectively. The rectangular metal patch on the top layer is mirrored symmetrically with the patch on the bottom layer. The metal layer in the middle layer is used as the ground layer between two substrates. Two via holes are drilled beside the metal patches on both sides along the *y*‐axis direction, passing through the ground plate without any connection, and another via hole is drilled beside the metal patch on the top layer connecting to the ground plate along the *x*‐axis. There are three PIN diodes embedded on the top and bottom layers of the metaparticle, and the equivalent circuits are exhibited in Figure 2c, in which the values of *C*, *L*, and *R* are 0.03 pF, 0.03 nH, and 7.8 Ω, respectively. A capacitance is loaded on the top side along the *y*‐axis to obstruct the direct current (DC) signal between the two metal patches. Under the illumination of the *x*‐polarized incident waves, the length of the rectangular metal patch along the *x*‐axis, *W*1, can affect the length of the surface current, thereby leading to the change of the resonant frequency of reflection. Meanwhile, the location of the via hole along the *x*‐axis, *L*1, can also determine the distribution of electric current on the metaparticle. Thus, by adjusting *W*1 and *L*1, their proper values can be selected to achieve the 1‐bit phase modulation in the reflection as desired. Similarly, *L*2 and *W*2 affect the amplitude and phase profiles in the transmission obviously. Besides, benefiting from the orthometric and symmetric edges of the rectangular patches, two current paths can be formed in orthometric directions under the *x*‐ and *y*‐polarized incident waves, which is good for the polarization isolation of the metaparticle. Other labeled geometrical parameters in Figure 2 are provided as *W* = 15 mm, *W*1 = 7.6 mm, *W*2 = 8.5 mm, *W*3 = 14.4 mm, *W*4 = 14.8 mm, *L*1 = 2.1 mm, *L*2 = 2.8 mm, and *H* = 1.5 mm.

**Figure 3:**
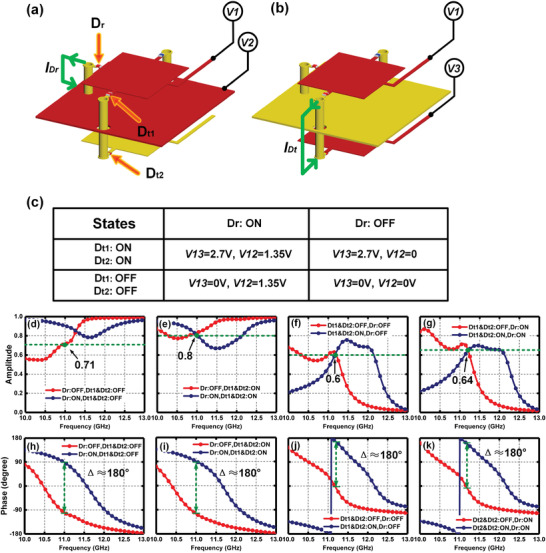
a,b) Simplified geometric structure of programmable metaparticle to illustrate the setups for applying the DC voltage used to control the state of Dt1, Dt2, and Dr independently. c) Details for the bias voltages applied to manipulate the PIN diodes. d–g) The simulated reflected and transmitted amplitude responses of the programmable metaparticle, when the Dt1, Dt2, and Dr are OFF and ON, respectively. h–k) The simulated reflected and transmitted phase responses of the programmable metaparticle, when the Dt1, Dt2, and Dr are OFF and ON, respectively.

The bias networks are etched on both sides of the metaparticle to supply the DC bias voltages controlled by FPGA. Two PIN diodes are loaded between the rectangle patches and via holes along the *y*‐axis, and another PIN diode is loaded between the rectangle patch and via hole along the *x*‐axis. Here, we define the PIN diodes on the top and bottom layers along the *y*‐axis as Dt1 and Dt2, and the PIN diode on the top layer along the *x*‐axis as Dr, respectively, as shown in **Figure** **3**a. By changing the bias voltages of two metal patches and ground, the working state of PIN diodes can be switched independently. In Figure 3a,b, we present the simplified geometric structures of metaparticle to exhibit the details of DC power supply networks. We define the DC voltage connected to the patch on the top layer, ground layer, and the patch on the bottom layer as *V*1, *V*2, and *V*3, respectively. When the difference between *V*1 and *V*2 reaches 1.32–1.45 V, the state of Dr is ON. Meanwhile, the direct current *I*_Dr_ is generated flowing from the top patch to the ground layer, as presented in Figure 3a. Similarly, when the difference between *V*1 and *V*3 is greater than 2.7 V, the direct current *I*_Dt_ will flow from the top patch to the bottom patch through the via hole along the *y*‐axis, and Dt1 and Dt2 will be ON, as shown in Figure 3b. The corresponding relationship between the DC bias voltages and state of Dr, Dt1, and Dt2 is exhibited in Figure 3c. It is observed that independent controls of the state of Dr, Dt1, and Dt2 can be realized benefiting from the separated current patches of *I*_Dr_ and *I*_Dt_, thereby leading to good isolation in the reflected and transmitted phase modulations. We should remark that Dt1 and Dt2 are placed in tandem, ensuring that they always share the same state in this design.

When Dt1 and Dt2 are switched ON (or OFF), the transmitted phase is encoded as T‐codes 1′ (or 0′). Similarly, the reflected phase is encoded as R‐codes 1 (or 0) corresponding to the state ON (or OFF) of Dr. In order to investigate reflected and transmitted properties of the metaparticle, we use commercial software, Computer Simulation Technology (CST) Microwave Studio, to simulate the amplitude and phase responses, in which the ON (or OFF) state of PIN diodes are represented by utilizing lumped elements in Figure 2c. In Figure 3d,h, the reflected amplitude and phase responses are illustrated under the *x*‐polarized incident plane waves when Dt1 and Dt2 are both in OFF. Obviously, the reflected phase difference between the 0 (red line) and 1 (blue line) is almost 180° and the amplitudes of reflections are greater than 0.71 around the frequency of 11 GHz. Figure 3e–i shows the simulated reflected amplitudes and phases in *x*‐polarization when Dt1 and Dt2 are both in ON. Comparing the simulated results in Figure 3d,e,h,i, it is clear that the reflected phases remain almost unchanged and the reflected amplitudes are larger than 0.7 at 11 GHz with different states of Dt1 and Dt2. Thus, we notice that the reflected phases and amplitudes are solely modulated by switching ON/OFF of Dr, and the influence of Dt1 and Dt2 can be ignored. Meanwhile, the simulated amplitude and phase responses of transmission are presented in Figure 3f,g,j,k with the illumination of *y*‐polarized plane waves. The difference of transmitted phases can attain 180° and the amplitude can reach more than 0.6 at 11.2 GHz. The effect of the state of Dr on the transmitted amplitude and phase is also negligible. From the simulated profiles in Figure 3, it is obvious that the proposed metaparticle has good orthogonal polarization isolations at the microwave frequency, and has the capability to modulate the transmitted and reflected phase responses in two orthogonal polarizations independently and simultaneously. Please refer to Note S1 in the Supporting Information for the electric‐field distributions of the metaparticle illuminated by the incident waves propagating along the −*z* direction under the *x*‐ or *y*‐polarization.

### 2.2 Performance of the Programmable Metasurface in the Reflection and Transmission modes

**Figure 4:**
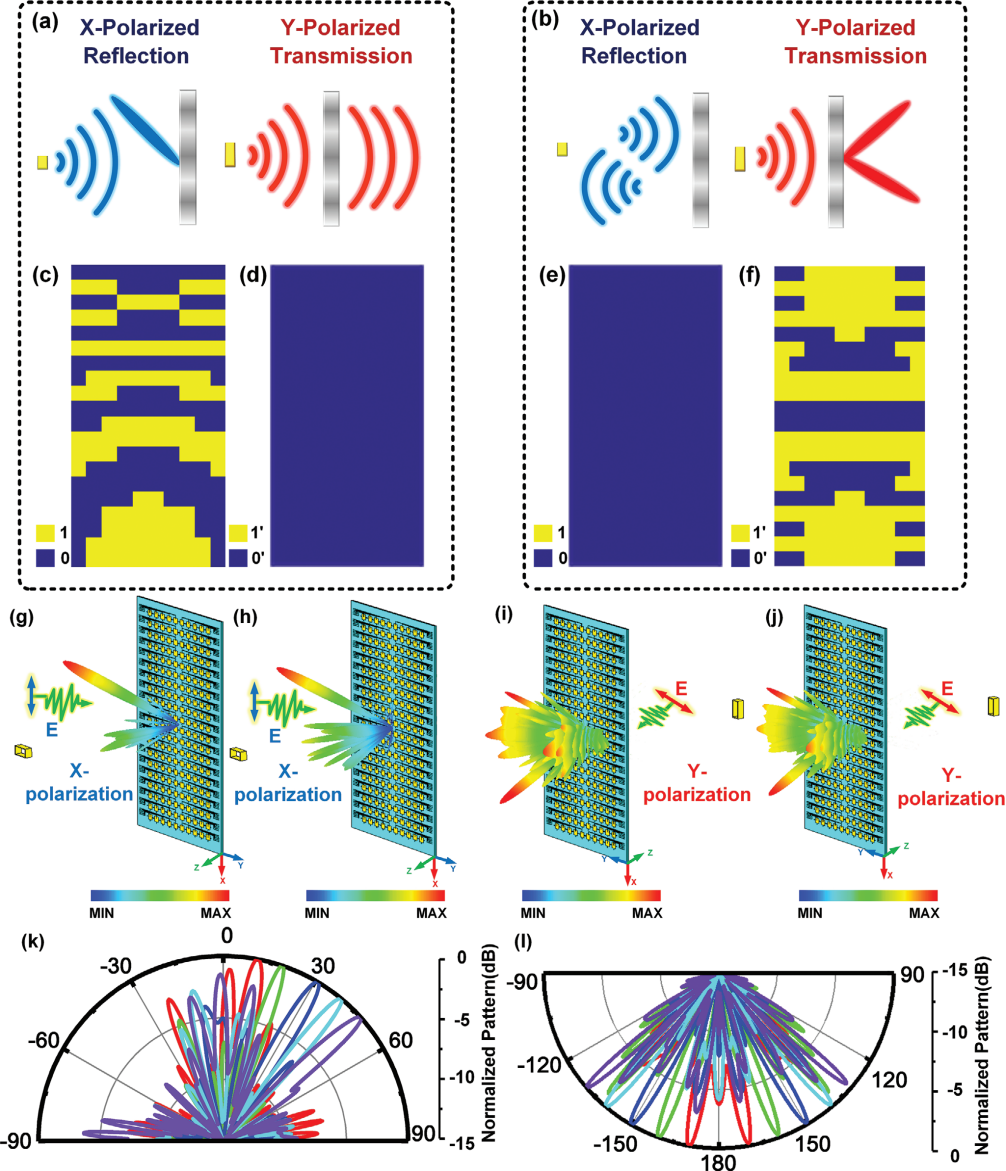
a) Schematic of programmable metasurface to control the reflected beam in *x*‐polarization. b) Schematic of programmable metasurface to control the transmitted beams in *y*‐polarization. c,d) The designed T‐coding and R‐coding patterns in (a) under *x*‐polarized incident wave propagating along −z direction. e,f) The designed T‐coding and R‐coding patterns in (b) under *y*‐polarized incident wave propagating along −z direction. g,h) 3D simulated reflected patterns of coding patterns in (c) and (d) at 11 and 11.4 GHz, respectively. i,j) 3D simulated transmitted patterns of coding patterns in (e) and (f) at 11.2 and 11 GHz, respectively. k) 2D simulated reflected patterns in the *ϕ* = 0° plane. l) 2D simulated transmitted patterns in the *ϕ* = 0° plane.

To validate the powerful ability of the proposed metaparticle to control the EM wavefronts on both sides, we design a programmable metasurface consisting of 10 × 20 metaparticles with a total size of 150 × 300 mm^2^ to attain the illustrative examples. First, we present a metasurface to modulate the reflected EM wavefronts without the transmitted modulations as shown in **Figure** **4**a, in which all transmitted phases behave as 0′ and the reflected beam is designed to generate at *θ* = 30° and *ϕ* = 0°. The coding patterns of reflection and transmission are presented in Figure 4c,d. A standard waveguide is adopted as the feeding source in this design, and the distance between the feeding source and metasurface is defined as *F*, where *F* is 250 mm. We show the reflected patterns in *x*‐polarization simulated by CST at 11 and 11.4 GHz in Figure 4g,h. It can be seen that the reflected beam appears approximately at *θ* = 30° in the *XOZ* plane in upper space, which coincides with theory and confirms the reflected performance of the programmable metasurface. Hence, when the R‐coding pattern is modulated diversely, the reflected beam in *x*‐polarization can be steered flexibly. In Figure 4k, we present the reflected 2D patterns simulated by CST at the *XOZ* plane, in which the reflected beam can steer from 10° to 50°, respectively.

Similarly, we propose another representative scheme for proving the properties of transmitted modulation, as illustrated in Figure 4b. The T‐coding pattern exhibited in Figure 4f is used to form two symmetrical beams, and the R‐codes in Figure 4e remains 0 at the same time. In Figure 4i,j, the corresponding results in full‐wave simulation of transmitted beams are provided at 11.2 and 11 GHz. The transmitted beams in *y*‐polarization are split into two beams symmetrically in the *XOZ* plane with *θ* = ± 30°. Moreover, we can manipulate the direction of symmetrical transmitted beams varying at ± 10°, ± 20°, ± 30°, ± 40° and ± 50° with different T‐code patterns, and the related 2D simulated patterns are shown in Figure 4l. In Figure 4g,j, it can be seen that some side lobes exist in the simulated patterns in the reflection and transmission modes, which are caused by the scattering from the metasurface. First, since the size of metasurface along the *y*‐axis is about only 5*λ* at 11 GHz, the ability for controlling the EM waves in the full space is limited. Then, when the incident waves illuminate the metasurface, the biasing lines will influence the distribution of the electric field and surface current, leading to the generation of side lobes in the simulation. For the transmitted patterns, a part of the incident waves coming from the feeding source propagates to the transmission without any modulation and is simulated as side lobes of the transmitted patterns in the far‐field. Thus, we can increase the quantity of the metaparticles on the designed programmable metasurface in the *y*‐axis to improve the performance of beams in the full space. Based on all simulated results in Figure 4, we believe the performance of the programmable metasurface in modulating the reflected (or transmitted) EM waves is verified separately and sufficiently. Since the reflected (or transmitted) coding patterns are related to the bias voltage controlled by FPGA, the functionalities of reflection (or transmission) can be dynamically adjusted as desired in real‐time.

**Figure 5:**
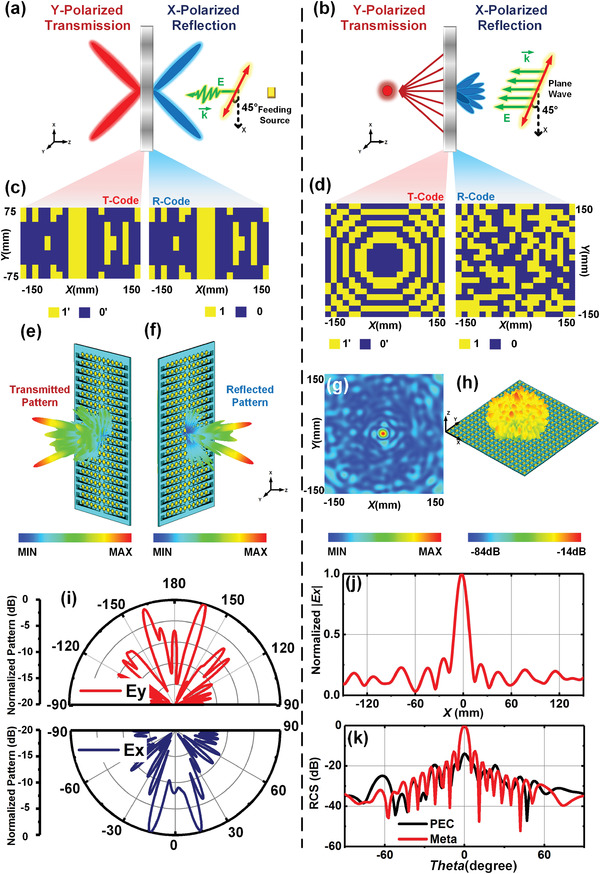
a) Schematic of programmable metasurface to control the reflected beam in *x*‐polarization and transmitted beam in *y*‐polarization with the incident wave obliquely polarized by a 45° angle with respect to the *x*‐axis. b) Schematic of programmable metasurface to generate a focal point in transmission and realize RCS reduction in reflection under illuminating of the incident plane waves obliquely polarized by a 45° angle with respect to the *x*‐axis. c,d) The designed T‐coding and R‐coding patterns in (a) and (b). e,f) The simulated transmitted and reflected pattern of patterns in (c) at 11 and 10.6 GHz in *y*‐polarization and *x*‐polarization, respectively. g,h) The simulated transmitted electric field in *y*‐polarization at *Zd* = 80 mm in *XOY* plane and reflected scattering pattern in *x*‐polarization. i) 2D simulated transmitted and reflected patterns of (e) and (f) in the *ϕ* = 0° plane. j) 2D simulated transmitted electric field intensity in the *XOY* plane at *Zd* = 80 mm with *Y* = 0 mm. k) The *x*‐polarized RCSs of the programmable metasurface and the metallic plane in reflection at 11 GHz.

In the above designed programmable metasurface, only the capability in controlling the reflected (or transmitted) wave is validated effectively. Here, considering the good orthogonal polarization isolations of the proposed metaparticle, we offer two schemes to examine the modulations of EM wavefronts on both sides at the same time, as exhibited in **Figure** **5**a,b. In Figure 5c, the designed T‐coding and R‐coding patterns on the programmable metasurface are illustrated, in which the T‐coding pattern is the same as the R‐coding pattern for generating two beams. It is remarkable to point out that a feeding source is placed in front of the metasurface with the *F* = 250 mm, and the incident wave coming from the source is obliquely polarized, where the angle between the direction of polarization and *x*‐axis is 45°. According to the mechanism of the polarization synthesis mentioned in Equation (2), the phase responses of metaparticle on two orthogonal components (*x*‐ and *y*‐polarizations) can be excited and modulated under the obliquely polarized incident waves simultaneously. Full‐wave simulation results of radiated patterns in transmission and reflection modes obtained by CST are presented in Figure 5e,f at 11 and 10.6 GHz, respectively. It can be clearly seen that the transmitted beams in the *y*‐polarization and reflected beams in the *x*‐polarization are both deflected into two beams symmetrically at *θ* = ± 16.5° and ± 15°. The corresponding 2D simulated patterns in the *XOZ* plane are shown in Figure 5i.

Besides, we know the coding patterns adopted in reflection and transmission can be different, benefiting from the separating DC path of metaparticle for controlling the state of PIN diodes embedded in the *x*‐axis and *y*‐axis. For the scheme depicted in Figure 5b, the programmable metasurface is designed to form a focal point on the *XOY* plane with a distance *Zd* = 80 mm in transmission and generate the diffuse reflection at 11 GHz. The T‐coding and R‐coding patterns are given in Figure 5d, in which the reflected phase is distributed randomly for reducing the radar cross‐section (RCS) in reflection. It is noticed that the designed programmable metasurface with patterns in Figure 5d is excited by obliquely polarized plane waves. In Figure 5g,h, we provide the simulated *y*‐polarized electric field intensity on the *XOY* plane with *Zd* = 80 mm and reflected scattering pattern in *x*‐polarization. It is obvious that the *x*‐polarized RCS reduction is 14 dB at least compared with the metallic reference plate causing by the destructive interference of the EM waves in reflection, as present in Figure 5k. Moreover, we demonstrate the 2D electric field intensity on the *XOZ* plane, and the EM wave in *y*‐polarization obviously converges at the center as anticipated. All the simulated results in Figure 5 suggest good agreement with our design and prove the ability of the programmable metasurface to manipulate the transmitted and reflected waves in two orthogonal polarizations simultaneously. Meanwhile, it is worth noting that the proposed programmable metasurface has the ability to control the deflected waves in any direction with desired *θ* and *ϕ* in the full space, and the related illustrative examples are presented in Note S2 in the Supporting Information. Thus, we believe that the proposed programmable metasurface can be used as a multifunction device by adjusting the bias voltage as designed, and these functionalities in full space can be realized and controlled in real‐time at the same time.

### 2.3 Experimental Verification

**Figure 6:**
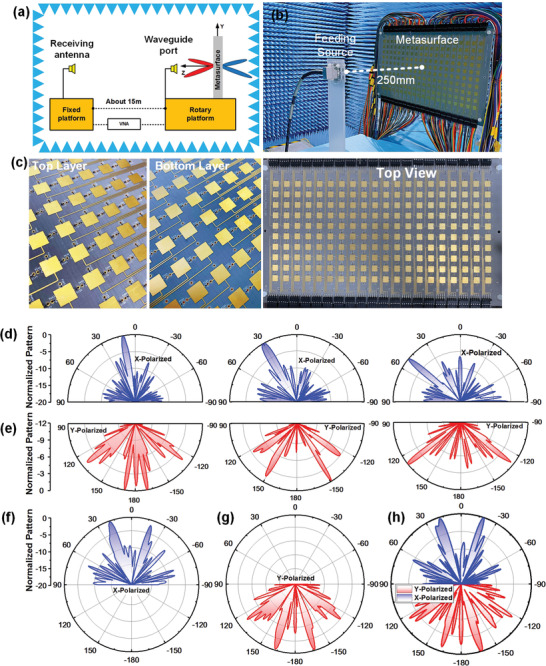
a,b) The experimental setup in an anechoic chamber. c) Photographs of the fabricated prototype. d) Measured reflected beams of three schemes in *x*‐polarization at 11 GHz, in which the different R‐code patterns are applied to form beams at *θ* = 10°, 30°, and 50°, respectively, with the T‐code patterns are all 0′. e) Measured transmitted beams of three schemes in *y*‐polarization at 11 GHz, in which the different T‐code patterns are applied to form two symmetrical beams at *θ* = ± 10°, ± 30°, and ± 50°, respectively, with the R‐code patterns are all 0. f) Measured reflected beams in *x*‐polarization and g) transmitted beams in *y*‐polarization of the T‐code and R‐code patterns in (c) under the illumination of *x*‐polarized and *y*‐polarized feeding source. h) Measured reflected beams (blue region) in *x*‐polarization and transmitted beams (red region) in *y*‐polarization of the T‐code and R‐code patterns in (c), when feeding waves are obliquely polarized by a 45° angle with respect to the *x*‐axis.

To experimentally validate the performance of the proposed programmable metasurface, we fabricate a prototype of programmable metasurface composed of 10 × 20 particles using the printed circuit board (PCB) technology. There are 3 × 10 × 20 PIN diodes employed on the programmable metasurface, with an overall size of 150 mm × 300 mm. All experiments in this paper are carried in a standard microwave anechoic chamber, and the experimental setup is shown in **Figure** **6**a,b. The fabricated sample and a waveguide are placed on a rotary platform, rotating in the horizontal plane covering 360°. A waveguide is fixed in front of the sample, acting as the feeding source with the excitation signal from 9.8 to 12 GHz. The distance between the feeding waveguide and the fabricated sample is 250 mm. The photographs of the fabricated sample and details of the biasing line are shown in Figure 6c. Considering the cost and complexity of fabrication, the biasing lines of every patch on the top and bottom layer are printed at the two sides of the sample, thereby leading to the independent control of bias voltages. Each column on the middle layer (composed of 10 metaparticles) is connected by biasing lines sharing a common control voltage. Therefore, the designed T‐coding and R‐coding patterns on the sample can be changed by switching the state of PIN diodes in real‐time.

In Figure 6d,e, the measured reflected patterns in *x*‐polarization at 11 GHz and transmitted patterns in *y*‐polarization at 11.2 GHz under the illumination of *x*‐ and *y*‐polarized incident waves are plotted, respectively, where the configurations of coding patterns are the same as these in Figure 4. It is clear that the reflected beams appear at 10°, 30° and 50°, and the transmitted beams are deflected into ± 10°, ± 30° and ± 50°. When the incident waves are in oblique polarization, the measured reflected pattern in *x*‐polarization and transmitted patterns in *y*‐polarization are exhibited in Figure 6h, where the T‐coding pattern and R‐coding pattern in Figure 5c are adopted on the sample. We can observe that the measured results in Figure 6h have good agreement with the simulated results in Figure 5i, thus validating the capability of the proposed programmable metasurface to modulate the radiated patterns on both sides simultaneously and independently in different polarizations. Furthermore, it is worth highlighting that there still are some side lobes and errors in the measured patterns due to the following reasons: 1) the errors of equivalent circuit models of PIN diodes and capacitances, which cannot fully represent the parameters in practical measurements. 2) The errors of manual operation and fabrication accuracy limitation of PCB technology. 3) In Figure 6b, it can be seen that some discrete wires on the fabricated programmable metasurface are used to connect the biasing line with FPGA, which will generate the scattering in the experiments. In the future, we can use the integrated socket instead of wires to improve the measured beams quality. Meanwhile, when the size of the programmable metasurface is large, it is noteworthy that the space on the top and bottom layers for printing the biasing lines will be limited. For solving this problem, we can transfer the biasing lines to the middle layer by via holes instead of printing on both sides, and the performance in the reflection and transmission of the metaparticle at 11 GHz can be maintained (see Figure S3 in the Supporting Information for details). On the other hand, the coupling between the biasing lines and metaparticles will be reduced when the biasing lines are printed on the middle layer, which can improve the performance of the measured patterns. Therefore, based on the above experimental results, the validity of the proposed programmable metasurface has been fully verified.

## 3 Conclusion

We have proposed a programmable metasurface to edit the reflected and transmitted waves simultaneously in the full space in real‐time at the microwave frequencies. By embedded three PIN diodes in the *x*‐axis and *y*‐axis, the digitized reflected and transmitted phase responses can be obtained. Benefiting from the design of the separated DC paths of PIN diodes, the working state of PIN diodes can be controlled independently by switching the bias voltage, thereby leading to independent controls of the reflected and transmitted phases in different polarizations. We encode the reflected phase as R‐codes, digits 0 and 1, and transmitted phase as T‐codes, digits 0′ and 1′, respectively. As a proof of principle, we put forward several coding schemes to precisely steer the beams in the reflection or transmission mode for verifying the properties of the proposed metasurface. More importantly, due to good isolation of polarizations, the reflected and transmitted waves can be excited and modulated as desired independently and simultaneously using different coding patterns when the incident wave is obliquely polarized. Therefore, while the bias voltages controlled by FPGA are switched dynamically, the designed functionalities in the full space can be implemented in real‐time. A sample is fabricated as proof to verify the validity of the design, and the measured results are coincident with the numerical simulations with good performance.

Compared with the reported passive metasurfaces offering the full‐space controls, the proposed programmable metasurface provides a flexible platform, on which the comprehensive controls of EM waves and various functions can be realized in a single platform, showing the bright prospects of the programmable metasurface. It is worth stressing that some challenges are still remained and need to be further investigated, such as the complex layout of biasing lines in large‐scale metasurface and the method for improving the precision of the equivalent circuits. We believe that the proposed programmable metasurface can provide more freedom in designing multifunctional and reprogrammable metadevices to dynamically control the EM waves in the full space by establishing independent controls of reflected and transmitted phase response. It can also promote more integrations between the programmable metasurface and intelligent components and systems.

## Conflict of Interest

The authors declare no conflict of interest.

## Supporting information

Supporting Information

## Data Availability

Data sharing is not applicable to this article as no new data were created or analyzed in this study.
