# Supplementary material for: Programmable Reflection–Transmission Shared‐Aperture Metasurface for Real‐Time Control of Electromagnetic Waves in Full Space
Source: Adv Sci (Weinh). 2021 May 26;8(15):2100149. doi: 10.1002/advs.202100149 (PMC8336522; doi:10.1002/advs.202100149)
Supplement: Supplementary file 1 — Supporting Information [file ADVS-8-2100149-s001.pdf]

© 2021 Wiley-VCH GmbH

ADVANCED SCIENCE
Open Access

Supporting Information

for *Adv. Sci.,* DOI: 10.1002/advs.202100149

Programmable reflection-transmission shared-aperture metasurface for real-time control of electromagnetic waves in full space

Lei Bao, Qian Ma, Rui Yuan Wu, Xiaojian Fu, Junwei Wu and Tie Jun Cui[*]

Supporting information:

Programmable reflection-transmission shared-aperture metasurface for real-time control of electromagnetic waves in full space

Lei Bao[1,2,3], Qian Ma[1,2,3], Rui Yuan Wu[1,2,3], Xiaojian Fu[1,2,3], Junwei Wu[1,2,3] and Tie Jun Cui[1,2,3*]

[1] Institute of Electromagnetic Space, Southeast University, Nanjing 210096, China

[2] State Key Laboratory of Millimeter Waves, Southeast University, Nanjing 210096, China

[3] Institute of Intelligent Metamaterials, Pazhou Laboratory, Guangzhou 510330, China

*Corresponding Author E-mail: tjcui@seu.edu.cn.

Note S1: The reflection and transmission schematics for the proposed programmable meta-particle, and the electric-field distributions of meta-particle under incident waves in x and y polarizations toward the –z direction.

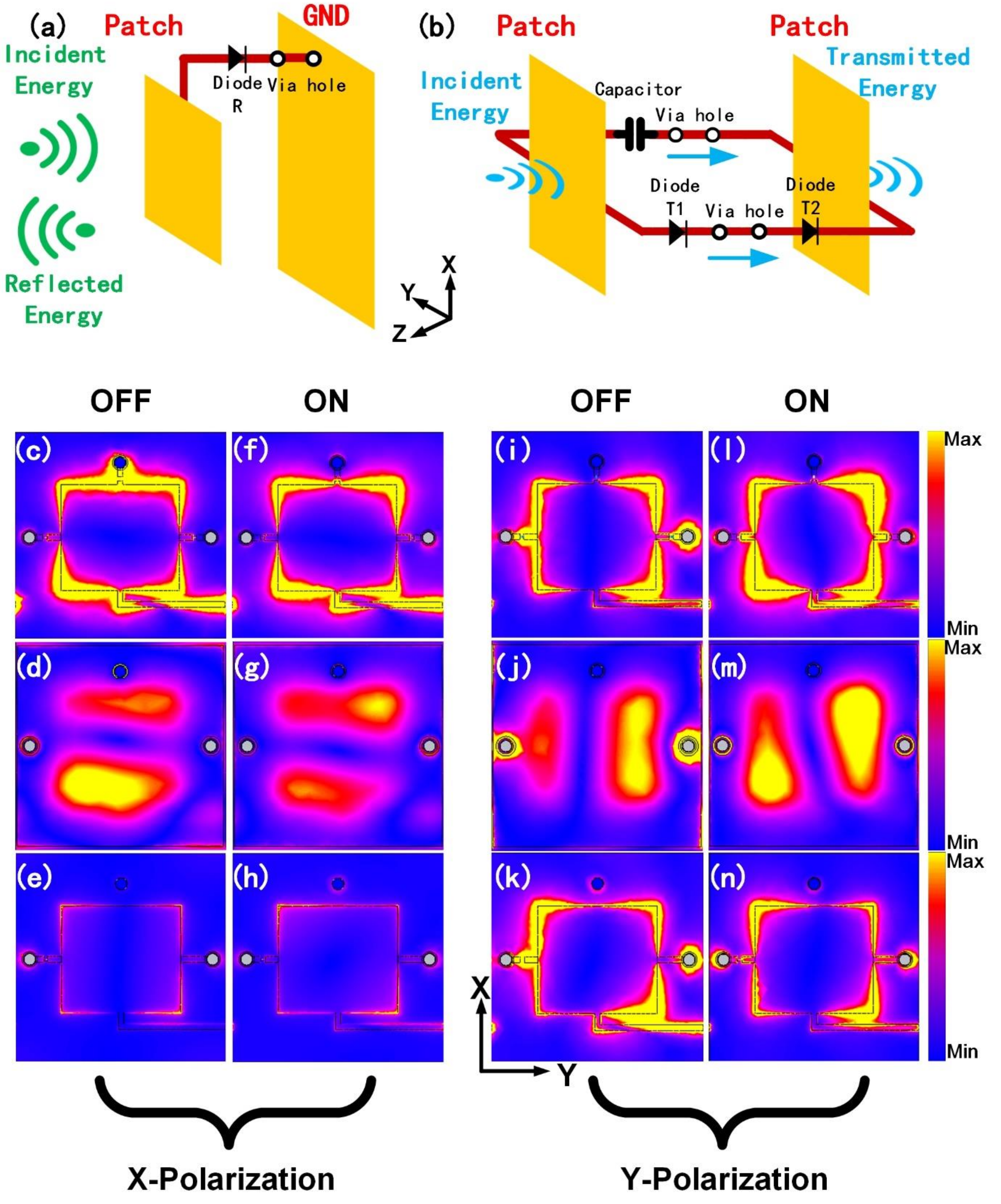

Figure S1. (a)-(b) The reflection and transmission schematics for the proposed programmable meta-particle. (c)-(e) and (f)-(h) The electric-field distributions of top layer, substrate, and bottom layer, under x-polarized

incident waves toward –z direction, when the Dr is OFF and ON, respectively. (i)-(k) and (l)-(n) The electric-field distributions of top layer, substrate, and bottom layer, under y-polarized incident waves toward –z direction, when the Dt1 and Dt2 are OFF and ON, respectively.

In **Figure S1a and S1b**, we provide the schematics of the energy transmission process in the reflection and transmission modes for further explaining the working mechanism of the meta-particle. When the x-polarized incident EM energy is captured by the patch on the top layer, the energy can be reflected by the ground layer and the reflected phase responses can be modulated through switching the state of Dr. For y-polarized spatial EM waves, the energy is firstly captured by the patch on the top layer. Then, the energy is transmitted to the patch on the bottom layer through via holes and PIN diodes along the y-axis and radiates back to space. **Figure S1c-S1n** illustrate the electric-field distributions of the meta-particle illuminated by the incident waves propagating along the -z direction. It can be seen that the electric field almost distributes on the top layer and ground layer under the illumination of x-polarized incident waves. When the incident waves are polarized along the y-axis, electric fields can penetrate through the ground layer and distribute on both metal patches of two sides. It is obvious that the transmitted path composed of two patches and via holes is open for y-polarized EM waves at 11.2 GHz. Consequently, we believe that the proposed meta-particle provides a new way to manipulate not only the transmitted wavefronts but also the reflected wavefronts on both sides of the metasurface with different polarized incidence.

Note S2: The illustrative examples of the proposed metasurface consisting of 20×20 meta-particles with a total size of $300 \times 300 mm^2$ for controlling the deflected waves in any direction with the desired θ and ϕ.

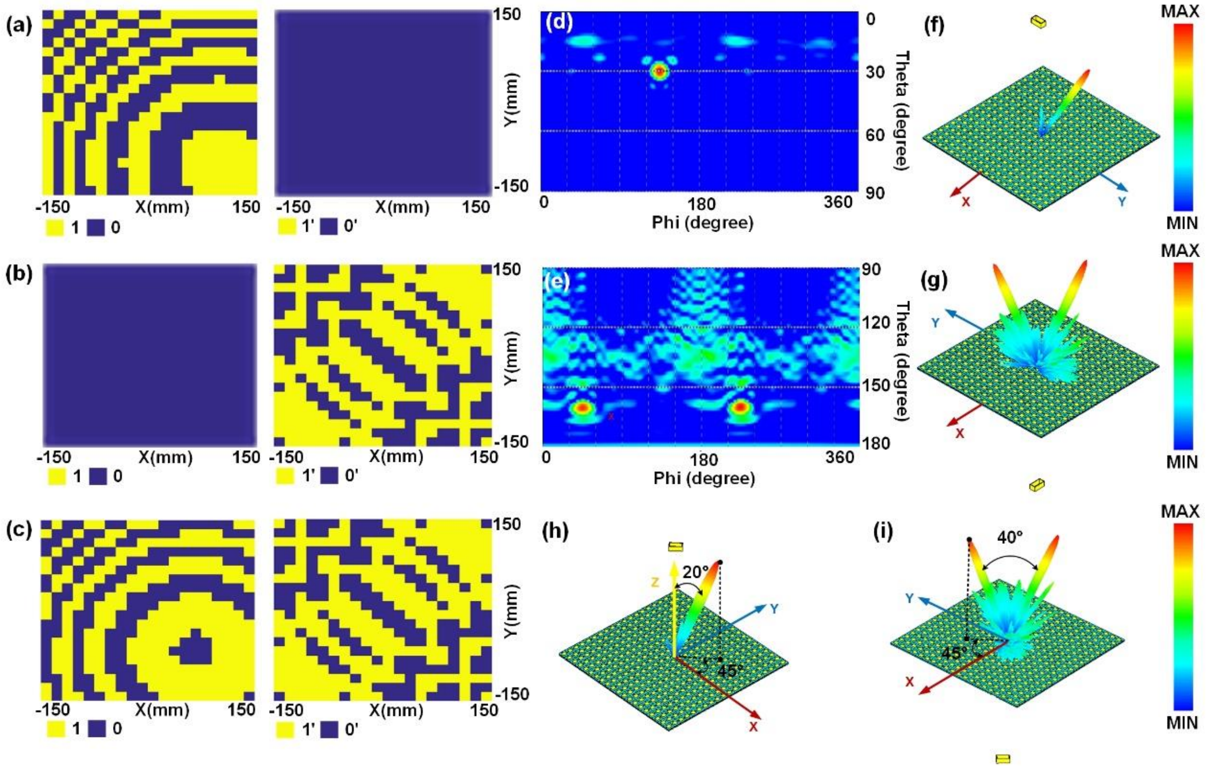

Figure S2. (a)-(c) The designed R-coding and T-coding patterns to generate the reflected and transmitted beams in (f), (g), (h) and (i). (d)-(e) The simulated 2D patterns in reflected and transmitted region at 11GHz and 11.2 GHz in x- and y-polarizations. (f) The simulated reflected 3D pattern of coding pattern in (a) with x-polarized incident waves. (g) The simulated transmitted 3D pattern of coding pattern in (b) with y-polarized incident waves. (h)-(i) The simulated 3D patterns in reflection and transmission of coding patterns in (c) under illuminating of the incident waves obliquely polarized by a 45° angle with respect to the x-axis.

In order to validate the ability of the metasurface to control the deflected waves in any direction with desired θ and ϕ, we provide a metasurface consisting of 20×20 meta-particles with a total size of $300 \times 300 mm^2$ to attain the several illustrative examples. The R-coding pattern exhibited in **Figure S2(a)** is used to form a reflected beam at θ= 30° and ϕ=45°, and

the T-codes in **Figure S2(a)** remains $0'$ at the same time. The corresponding results in full wave simulations of reflection are shown in **Figure S1(d) and (f)**, respectively. It is clear that the reflected beam appears at θ= 30° and ϕ=45° in the upper space. Similarly, we also present another scheme for forming two symmetrical beams at ϕ =45° and 225°. The angle between the two simulated beams is 40° at 11.2 GHz. Finally, we offer a scheme to generate a reflected beam and two transmitted beams simultaneously, and the incident wave coming from the source is obliquely polarized, where the angle between the direction of polarization and the x-axis is 45°. The corresponding coding patterns are shown in **Figure S1(c)**. The simulated results of radiated patterns are provided in **Figure S2(h)-(j)**, in which the reflected beam generates at θ= 20°, ϕ=45° in x-polarization at 11GHz, and the transmitted beams generate at θ= 20°, ϕ=45° and θ= 20°, ϕ=225° in y-polarization at 11.2GHz. Based on all simulations in **Figure S2**, we believe that the proposed programmable metasurface can deflect the reflected or transmitted beam in any direction with the desired θ and ϕ.

Note S3: The performance of the meta-particle with via holes on the biasing lines.

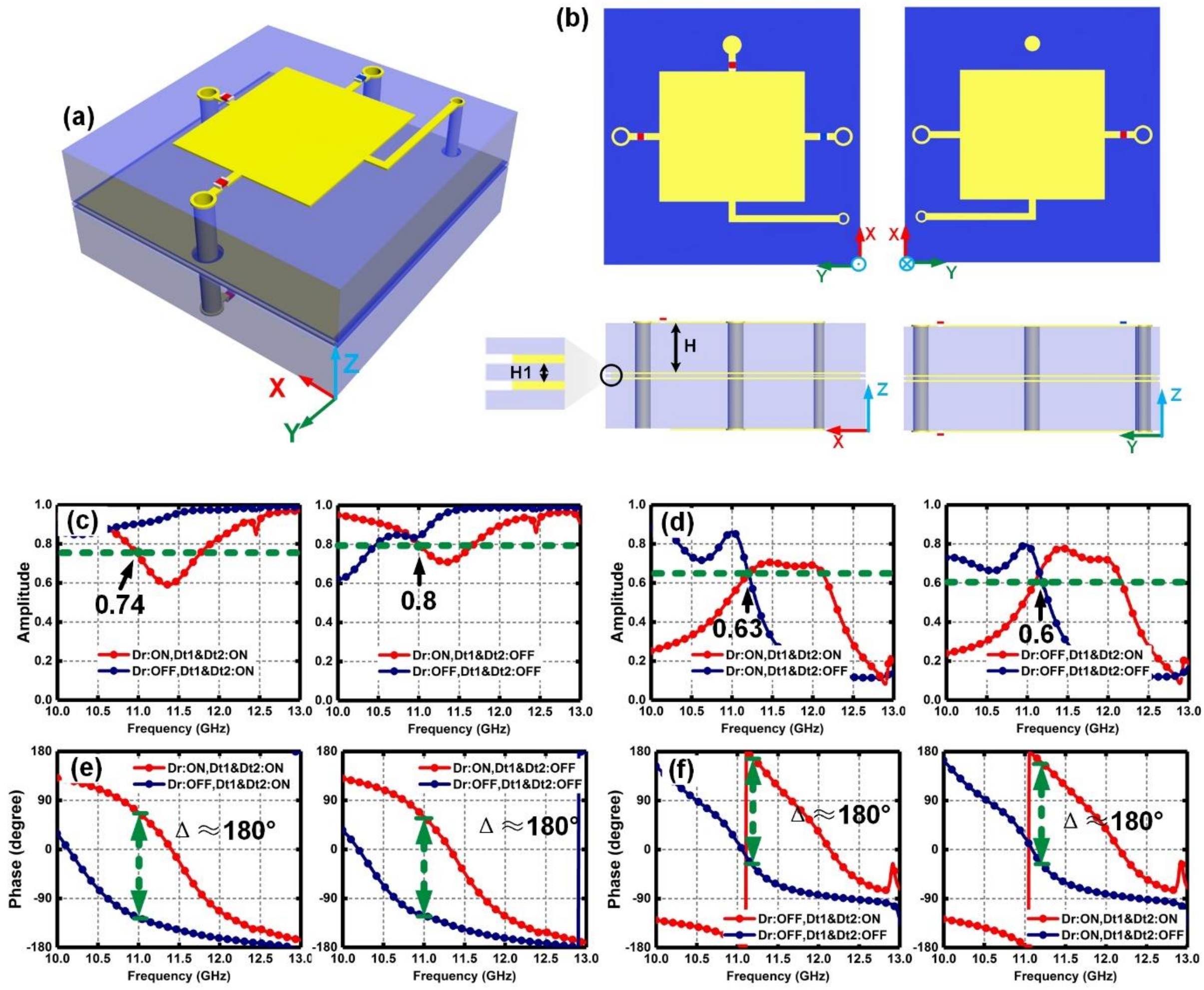

Figure S3. (a)–(b) Geometry of the programmable meta-particle with via holes on biasing lines. (c)-(d) The simulated reflected and transmitted amplitude responses of the programmable meta-particle, when the Dt1, Dt2, and Dr are ON and OFF, respectively. (e)-(f) The simulated reflected and transmitted phase responses of the programmable meta-particle, when the Dt1, Dt2, and Dr are ON and OFF, respectively.

In **Figure S3**, we adopt another designed scheme of the biasing lines, in which the biasing lines printed on the top and bottom layers are transferred to the middle layer through via holes as shown in **Figure S3(a)**. In **Figure S3(b)**, a substrate layer is put between two GND layers, and the thickness is 0.2mm (*H1*=0.2mm). Other geometrical parameters of the metal-particle remain unchanged and *H*=1.4mm. By utilizing the technique of PCB, a large of biasing lines can be designed and printed on the middle substrate layer. In order to ensure the performance of

the meta-particle, we have simulated the amplitude and phase responses in reflection and transmission of the meta-particle designed in **Figure S3**, and the results have been exhibited in **Figure S3(c)-(f)**. Comparing the profiles with these in **Figure 3**, we can find that the parameters of the meta-particle in reflection and transmission are not influenced and almost remain unchanged. So, this scheme of supply networks can be used to the programmable metasurface with large size.
